# Lamotrigine-induced hemophagocytic lymphohistiocytosis with Takotsubo cardiomyopathy: a case report

**DOI:** 10.1186/s13256-019-2295-1

**Published:** 2019-11-26

**Authors:** Jenny Y. Zhou, Jordan A. Martinez, John Paul Shen

**Affiliations:** 1 Department of Medicine, University of California, San Diego, San Diego, CA USA; 2 Department of Gastrointestinal Medical Oncology, University of Texas MD Anderson Cancer Center, Houston, TX USA

**Keywords:** Hemophagocytic lymphohistiocytosis, Lamotrigine, Medication adverse effect, Takotsubo cardiomyopathy

## Abstract

**Background:**

Hemophagocytic lymphohistiocytosis is a rare hematological syndrome characterized by excessive and uncontrolled activation of the immune system. The often nonspecific nature of early symptoms and the potential for progression to multiorgan failure and death if appropriate therapy is not started promptly, highlight the importance of heightened recognition for this uncommon disease. Although there are well-described associations of hemophagocytic lymphohistiocytosis with infectious, malignant, and autoimmune diseases and an established treatment protocol for these cases, the link between medications and hemophagocytic lymphohistiocytosis is less clearly established and the optimal treatment of these cases less well defined.

**Case presentation:**

Here we describe the case of a 45-year-old caucasian woman presenting with signs and symptoms consistent with hemophagocytic lymphohistiocytosis, induced by recent exposure to lamotrigine. She had a rapidly progressive clinical course, complicated by multiorgan failure including stress-induced Takotsubo cardiomyopathy and cardiac arrest. With dexamethasone and etoposide therapy, she made a full and sustained recovery.

**Conclusions:**

This case highlights that medication-induced hemophagocytic lymphohistiocytosis appears to respond similarly to the same dexamethasone and etoposide treatment regimen developed for other non-drug-induced forms of hemophagocytic lymphohistiocytosis. With the continued cessation of the offending agent there has not been need for maintenance therapy and no relapse to date. In addition, given the risk for cardiomyopathy, a clinical complication not classically associated with hemophagocytic lymphohistiocytosis, echocardiogram and telemetry monitoring should be considered in the initial workup of suspected hemophagocytic lymphohistiocytosis.

## Background

Hemophagocytic lymphohistiocytosis (HLH) is an uncommon but dire consequence of uncontrolled activation of the immune system. Previously, HLH has been primarily associated with pediatric populations; however, over the past 10 years it has been increasingly identified in adult patients with the exact incidence unknown [1]. There are both primary, due to germline genetic mutations, and secondary forms of HLH. Secondary HLH is more common in adults and can be triggered by various states of relative immune activation (malignancy, autoimmune disease, infection) and less commonly by medications [2]. These triggers are thought to activate macrophages which phagocytize blood cells and release large amounts of pro-inflammatory cytokines. The resulting cytokine storm causes a wide range of end-organ damage and can be rapidly fatal if not diagnosed and treated promptly [1, 2]. Diagnosis is generally based on defined clinical criteria from the HLH-2004 trial which requires five of eight of the following manifestations: fever, cytopenias affecting two more lineages, splenomegaly, hypertriglyceridemia, hypofibrinogenemia, hyperferritinemia, low or absent natural killer (NK) cell activity, and elevated soluble CD25 [3]. Additional findings include transaminitis, coagulopathy, edema, rash, and neurologic symptoms [3–5].

There are a number of conditions associated with secondary HLH. By prevalence these include malignant (50–60%), infectious (25–34%), autoimmune (3–8%), and idiopathic causes [4, 5]. Although certain medications have also been linked to HLH, these connections are less clearly established and to date only reported in case reports or case series [6, 7].

In this case report, we present a previously health woman who developed a rapidly progressive HLH course triggered by the medication lamotrigine. With prompt cessation of this causative agent as well as initiation of HLH-directed therapy, she made a full and, to date, sustained recovery. This case highlights the importance of heightened clinical suspicion for HLH secondary to medications, the potential for HLH to involve any organ system (in this case Takotsubo cardiomyopathy), and that these multiorgan effects can be reversed with appropriate HLH therapy.

## Case presentation

A 45-year-old caucasian woman with past medical history significant for generalized anxiety and major depression disorder presented with influenza-like symptoms including fevers and neck stiffness of 1-week duration. She worked as an accountant and had no tobacco smoking or alcohol history. Her family history was significant for depression in multiple members as well as stroke in a grandmother and gastric cancer in a grandfather. She is married with two children, who are healthy. Home medications consisted of amlodipine, cholecalciferol, clonazepam, duloxetine, and prazosin, and lamotrigine which was started 17 days prior to admission for recurrent major depression. She had been on all her other medications for years. Lamotrigine was held upon admission. Initial laboratory data were significant for: acute anemia with hemoglobin (Hgb) of 10.8 gm/dL; thrombocytopenia (platelet count of 95,000/mm^3^); transaminitis with aspartate aminotransferase (AST) of 151 U/L, alanine aminotransferase (ALT) of 59 U/L, alkaline phosphatase of 164 U/L with initial normal bilirubin level; elevated inflammatory markers with ferritin of 29,101 ng/mL, lactate dehydrogenase (LDH) of 1101 U/L, and D-dimer of 62,365 ng/mL; and normal white count. Initial vital signs revealed fever of 38.8 °C and tachycardia of 120 beats per minute with normal blood pressure of 110/64. A physical examination revealed a mildly anxious woman with no focal neurologic deficits, sinus tachycardia, clear lung fields, no palpable splenomegaly or lymphadenopathy, and a maculopapular rash on her trunk and bilateral lower extremities. Imaging included a chest X-ray which showed no obvious pneumonia and abdominal ultrasound (US) which showed hepatomegaly with increased echogenicity thought to be fatty liver and spleen size of 11–12 cm. A computed tomography (CT) scan of her chest was also done which revealed bibasilar opacifications thought to be atelectasis and small bilateral pleural effusions. A lumbar puncture performed in the Emergency Department had a normal cell count, protein level of 38 mg/dL, and glucose of 60 mg/dL with negative cerebral spinal fluid (CSF) bacterial culture and meningitis panel.

**Fig. 1:**
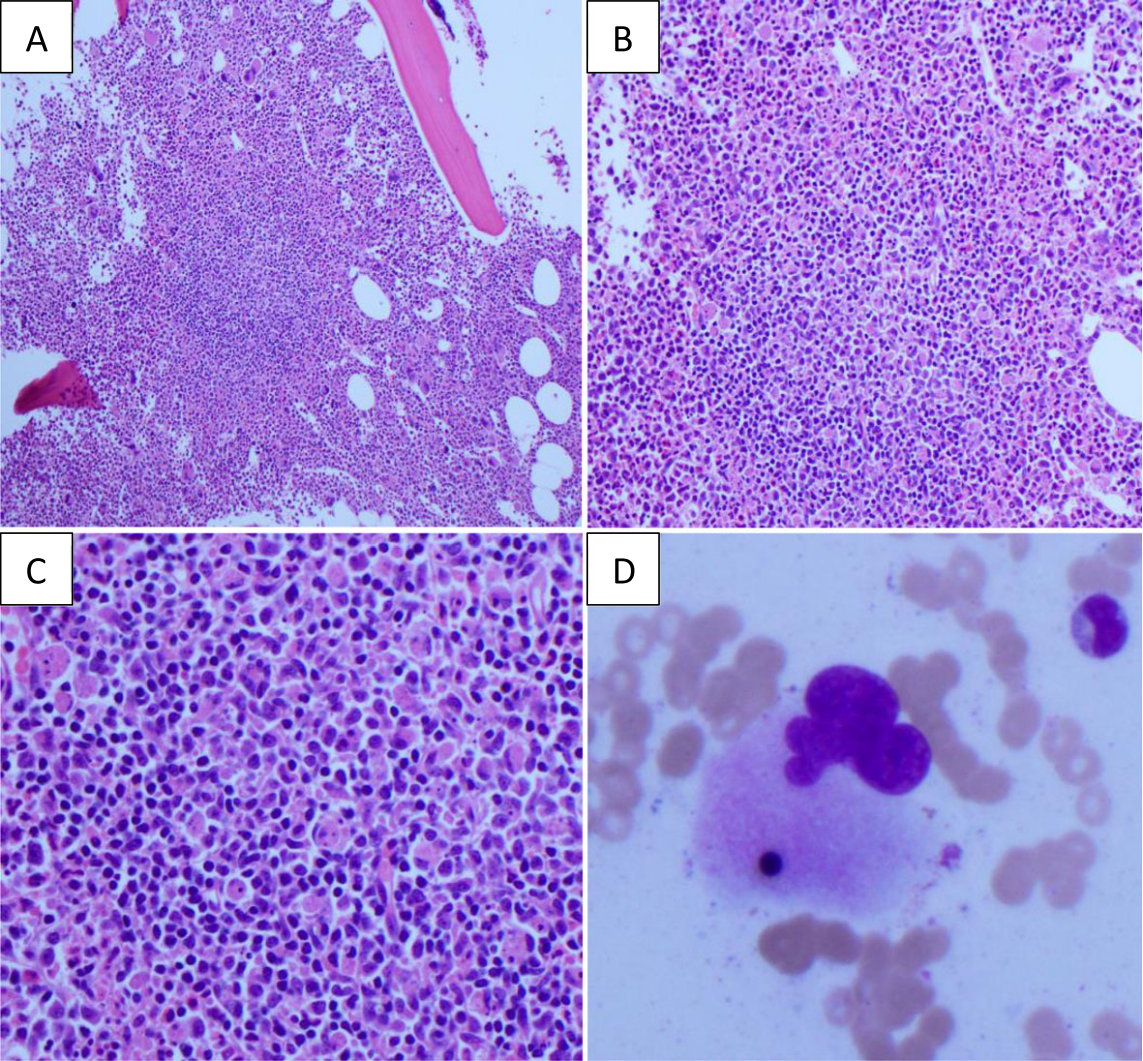
Bone marrow biopsy. **a**–**c** Hypercellular bone marrow with trilineage hyperplasia and lymphohistiocytic aggregates with increased phagocytic activity. **d** Macrophage with phagocytosed erythroid precursor

She was admitted for management of sepsis and acute hypoxic respiratory failure. She was started on broad spectrum antibiotics with intravenously administered vancomycin, ceftazidime, and metronidazole, which she remained on for 4 days. Hematology was consulted for evaluation of acute cytopenias and coagulopathy. Further studies included a peripheral blood smear which was notable for band neutrophils with prominent toxic granulation, anisocytosis, and poikilocytosis but normocytic red blood cells, thrombocytopenia with normal platelet morphology, and no schistocytes or fragmented red blood cells to suggest a microangiopathic process. An extensive infectious disease and autoimmune workup was also initiated. Given the constellation of findings that included severe hyperferritinemia > 50,000 ng/mL, coagulopathy with elevated prothrombin time (PT)/partial thromboplastin time (PTT), up-trending liver function tests (LFTs), and low fibrinogen with markedly elevated D-dimer and LDH, there was a high suspicion for an excessive immune activation process such as HLH. A bone marrow biopsy was performed (Fig. 1) and other HLH studies including serum triglycerides (which was initially mildly elevated at 185 mg/dL but quickly up-trended to > 3000 mg/dL), NK cell activity (by flow cytometry which was normal), and soluble interleukin-2 (IL-2) receptor, and a germline mutation panel was sent.

**Fig. 2:**
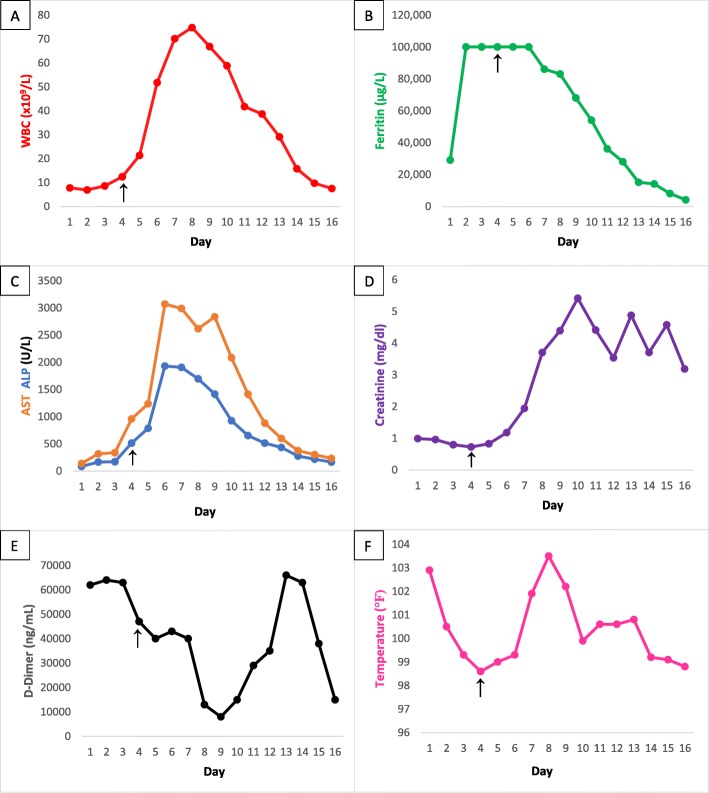
Hemophagocytic lymphohistiocytosis markers and response to treatment. *Arrow* indicates when treatment with the HLH-94 protocol was initiated. **a** White blood cell count. **b** Inflammatory marker ferritin (note, 100,000 is the maximal quantitative value, days 2–6 had values documented as > 100,000). **c** Liver function tests, aspartate transaminase and alkaline phosphatase. **d** Creatinine levels. Intermittent dialysis was initiated on day 10. **e** Daily maximum temperature. **f** Inflammatory marker D-dimer (highly sensitive assay). *ALP* alkaline phosphatase, *AST* aspartate transaminase, *WBC* white blood cell

As her clinical course evolved with rapidly rising ferritin (> 100,000 ng/mL) and worsening LFTs and creatinine, as well as the development of multiorgan failure (Fig. 2**)** requiring transfer to a monitored unit, a decision was made to start empiric treatment for HLH per the HLH-94 protocol [8, 9]. The protocol entails an 8-week induction therapy of etoposide (150 mg/m^2^ twice weekly for 2 weeks and then weekly) and dexamethasone (initially 10 mg/m^2^ for 2 weeks followed by 5 mg/m^2^ for 2 weeks, 2.5 mg/m^2^ for 2 weeks, 1.25 mg/m^2^ for 1 week, and 1 week of tapering) [8]. Intrathecal methotrexate is given for patients with suspected central nervous system (CNS) involvement. At the time of the decision to start HLH-directed therapy, bone marrow biopsy results were available showing a hypercellular marrow with no evidence of hematologic malignancy but two foci of hemophagocytosis (Fig. 1). In addition, an infectious disease workup did not identify a likely cause of fever, including extensive viral, bacterial, and fungal tests which included blood and urine cultures, gastrointestinal (GI) pathogen panel, herpes simplex virus (HSV) polymerase chain reaction (PCR), human immunodeficiency virus (HIV) PCR, cytomegalovirus (CMV) PCR, coccidioidomycosis, *Histoplasma*, QuantiFERON, *Cryptococcus*, and parvovirus serologies which were all negative. Of note, Epstein–Barr virus (EBV) PCR was detectable at 6633 deoxyribonucleic acid (DNA) IU/mL; however, the infectious disease specialists felt this to be more reflective of asymptomatic viremia in the setting of an excessive inflammatory state rather than the trigger. Furthermore, EBV staining in the bone marrow was negative by *in situ* hybridization. An autoimmune workup was also unremarkable and CT imaging revealed no overt malignancy.

The morning after dexamethasone was started our patient experienced a witnessed ventricular fibrillation arrest. The Code Blue (critical medical emergency) team was activated and was able to achieve a return to spontaneous circulation, during the Code Blue our patient was intubated for airway protection. Her intensive care unit (ICU) course was complicated by acute renal failure requiring intermittent dialysis, progressive transaminitis, and an acute drop in left ventricular ejection fraction with a transthoracic echocardiogram showing Takotsubo cardiomyopathy. HLH-directed therapy as per HLH-94 regimen [8, 9] was continued while she was intubated and in the ICU. She began to show clinical improvement 2 days after the first etoposide dose, and a gradual decline in ferritin, white blood cell (WBC) count, and liver enzymes began at this time as well (Fig. 2). She continued to show clinical improvement, allowing for extubation and downgrade from the ICU 1 week after initiation of HLH therapy. Improvement in renal dysfunction was delayed relative to liver enzymes and WBC count, requiring the start of hemodialysis as well as dose reduction in etoposide; however, her renal function eventually returned to baseline with discontinuation of dialysis after 8 days. Soluble IL-2 receptor which had been elevated to 10,270 pg/mL initially (reference range < 1033 pg/mL) also returned to normal.

She was able to complete the remainder of the 8-week HLH-94 protocol, of note, no intrathecal methotrexate and hydrocortisone was given as there was no evidence of CNS involvement [9]. Laboratory parameters continued to improve including resolution of her significant hyperferritinemia, transaminitis and leukocytosis. Her cardiac function also normalized with ejection fraction improvement to 70% from 36% after her cardiac arrest. At time of discharge after a hospital course that spanned 44 days, she was referred to a Bone Marrow Transplant clinic for consideration of allogeneic transplant. At that time, germline mutation testing came back showing no known pathogenic variants. Due to her complete response and sustained resolution of symptoms currently 9 months since time of admission, a transplant has not been deemed to be indicated, although she is being closely monitored at regular intervals for any signs of recurrence. Lamotrigine had been held since her initial presentation.

Clinical data are from University of California San Diego clinical laboratories; germline sequencing for hereditary HLH-associated genes (*ADA, AP3B1, BL0C1S6, BTK, CD27, IL2RA, IL2RG, ITK, LYST, MAGT1, MVK, PNP, PRF1, RAB27A, SH2D1A, SLC7A7, STX11, STXBP2, UCN13D, WAS, XIAP*) was performed at ARUP Laboratories (Salt Lake City, UT, USA).

## Discussion and conclusions

In this case report, we describe a woman diagnosed as having HLH presumably induced by lamotrigine given the temporal association with this medication and the exclusion of all other potential etiologies. She was started on lamotrigine, an antiepileptic medication commonly prescribed for major bipolar depression, 17 days prior to her presentation, which quickly evolved into multiorgan failure with cardiac arrest, and renal and liver failure. After initiation of HLH-directed therapy and cessation of lamotrigine, she fully recovered. Recently, the US Food and Drug Administration (FDA) release a safety announcement in April 2018 to warn that lamotrigine can cause a rare but severe immune system reaction meeting diagnostic criteria for HLH [10]. Eight known cases had been identified by the time of this release; we have now presented a ninth [10, 11]. HLH is characterized by uncontrolled inflammation and immune system activation often with devastating consequences. In acquired HLH, an immunologic trigger, usually infection, malignancy, or autoimmune disease [12, 13], activates macrophages and a cytokine storm promotes down-regulation of cell surface markers that prevent phagocytosis. The inflammatory milieu is responsible for prolonged fevers and malaise while promoting ingestion of precursors of all the major hematopoietic cell lines in the bone marrow [2].

In this case the inciting event was the drug lamotrigine, given the clear temporal association of HLH following the start of the medication and exhaustive exclusion of other etiologies. Lamotrigine is commonly used as an antiepileptic as well as for maintenance treatment of bipolar I disorder; in total, it is prescribed more than 12 million times a year in the USA [14]. Recently, the FDA released a warning of eight confirmed cases worldwide of lamotrigine-triggered HLH, this case is the ninth overall and the first case of HLH associated with cardiomyopathy and arrest [10]. While medications have been previously linked to secondary HLH, drug-induced HLH is less common than other causative etiologies [6, 7] and importantly there is no mention of the inclusion of such patients in the HLH-94 trial which established the standard-of-care treatment for this disease [8]. We suspect the underlying pathophysiology relates to a drug hypersensitivity reaction triggering an excessive activation of macrophages with the resultant hyperinflammatory sequelae; however, with only one prior case report of lamotrigine-induced HLH [11] data are lacking to identify more specific mechanisms. Importantly, this patient made a complete recovery after being treated with dexamethasone/etoposide induction therapy and has not required maintenance therapy. Although our patient had several favorable clinical factors, such as no HLH-associated germline mutations, age under 50 years, no organ dysfunction at baseline, and a short time to treatment [2], her favorable outcome and that of a prior report [11] suggest that lamotrigine-induced HLH may have a better long-term prognosis relative to other secondary forms of HLH, provided the patient is not re-exposed to the offending agent.

Our patient’s clinical course was complicated by stress-induced (Takotsubo) cardiomyopathy [15], a condition previously associated with HLH in three case reports [16, 17]. However, to the best of our knowledge this is the first documented case of cardiac arrest secondary to ventricular fibrillation with Takotsubo cardiomyopathy in the setting of HLH. Our patient’s ejection fraction recovered within 1 week of treatment initiation, and the remainder of her cardiac course was only significant for premature ventricular contractions deemed secondary to resolving HLH. Given the difficulty in rapidly and correctly diagnosing HLH, performing an echocardiogram to look for signs of Takotsubo cardiomyopathy is a rapid and non-invasive test that can aid in diagnosis if positive. Given the risk for cardiac arrest, providers should have a low threshold to initiate telemetry monitoring for patients with suspected HLH.

## Conclusion

In summary, while secondary HLH is usually triggered by infection or malignancy, a thorough medication history is essential for prompt recognition and cessation of potentially offending agents such as lamotrigine. Outcomes for such patients with withholding of offending drug and prompt initiation of dexamethasone/etoposide treatment, which can be started empirically (while specialized laboratory results are pending) if other HLH-94 criteria are met, seems favorable and similar to non-drug-induced forms of secondary HLH. In addition, given the risk for cardiomyopathy, a clinical complication not classically associated with HLH, echocardiogram and telemetry monitoring should be considered in the initial evaluation of HLH.

## Data Availability

Data sharing is not applicable to this article as no datasets were generated during the current study.

## Abbreviations

ALT: Alanine aminotransferase
AST: Aspartate aminotransferase
CMV: Cytomegalovirus
CNS: Central nervous system
CSF: Cerebral spinal fluid
CT: Computed tomography
DNA: Deoxyribonucleic acid
EBV: Epstein–Barr virus
FDA: Food and Drug Administration
GI: Gastrointestinal
Hgb: Hemoglobin
HIV: Human immunodeficiency virus
HLH: Hemophagocytic Lymphohistiocytosis
HSV: Herpes simplex virus
ICU: Intensive care unit
IL-2: Interleukin-2
LDH: Lactate dehydrogenase
LFTs: Liver function tests
NK: Natural killer
PCR: Polymerase chain reaction
PT: Prothrombin time
PTT: Partial thromboplastin time
US: Ultrasound
WBC: White blood cell
